# Measles outbreaks – potential threat for health care professionals

**DOI:** 10.1016/j.infpip.2020.100074

**Published:** 2020-07-09

**Authors:** A.C. Westgeest, D. de Mooij, C.Y. Eger, N.M. Delfos, M. van der Feltz, L.G. Visser, G.H. Groeneveld

**Affiliations:** a Leiden University Medical Centre, Department of Infectious Diseases, C5-P, P.O. box 9600, 2300 RC Leiden, The Netherlands; b Alrijne Hospital Leiderdorp, Simon Smitweg 1, 2353 GA Leiderdorp, The Netherlands

**Keywords:** Measles, Nosocomial infection, Health care professionals

## Abstract

The ongoing spread of measles is a major concern for public health. Optimal vaccination coverage amongst health care professionals (HCP) is essential for individual protection. This is illustrated by our two cases of measles infection in HCP during the 2018 outbreak in Europe.

We developed a questionnaire to assess protection against measles amongst HCP working in acute care of a tertiary hospital in The Netherlands. In total, 29% of these professionals were not protected against measles. During current worldwide measles outbreaks, it is paramount for employee health protection, patient protection and disease control to register and optimize employees' immunity.

## Introduction

The ongoing spread of measles throughout Europe in 2018 [1] and the outbreak in Rockland County [2] are major concerns for public health. The suboptimal vaccination coverage enables outbreaks and further spread. Health care settings are potential transmission sites and may pose health care professionals at risk. Instant recognition of measles is paramount to institute adequate (airborne) isolation. Optimal vaccination coverage amongst HCP is essential for HCP and patient protection.

We present two cases of measles in HCP during the 2018 outbreak in Europe and discuss protection rates amongst HCP in acute care.

## Cases

A 50-year old woman presented to the emergency department of hospital A with clinical signs and symptoms of measles. She was a nurse at the acute admission department of hospital B, a tertiary care hospital. She thought she had had measles as a child. During her childhood, measles vaccination was not yet included in the national vaccination program.

**Figure 1:**
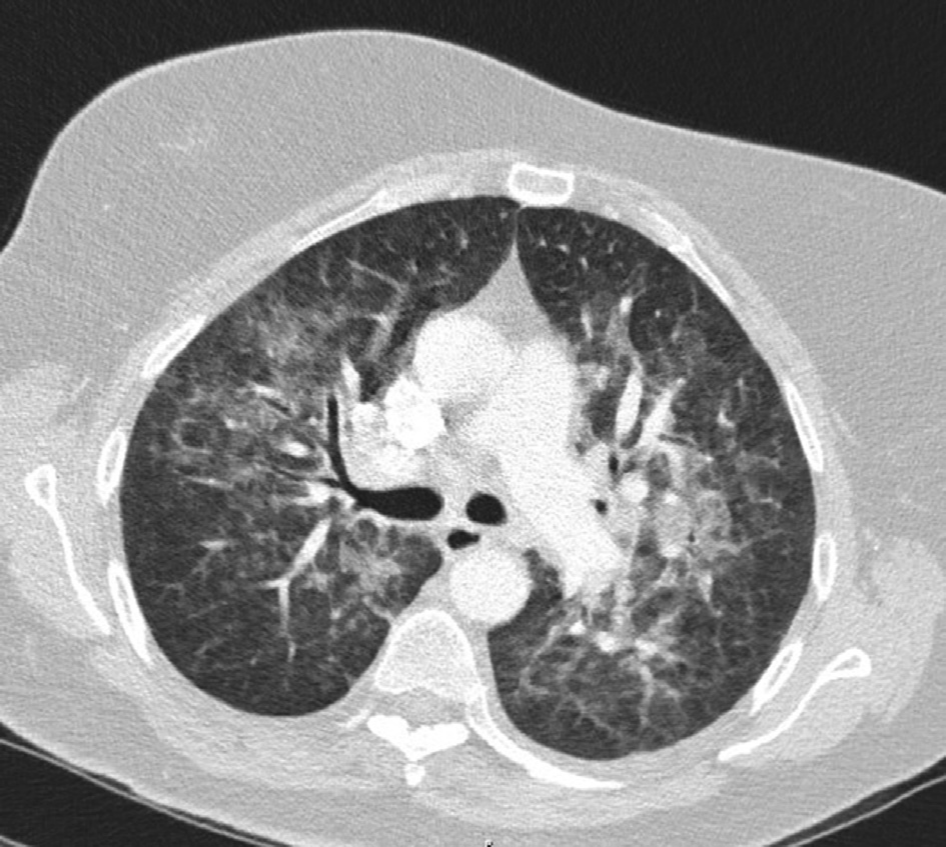
CT imaging of the thorax of patient 1 showing interstitial pulmonary infiltrates.

Within 24 hours after admission, she developed respiratory failure. CT imaging of the thorax showed interstitial pulmonary infiltrates (Figure 1). Measles pneumonia was confirmed by positive PCR in bronchial fluid and positive serum IgM. Eight days later she was discharged from the intensive care unit but it took several months before she fully recovered.

Retrospective questioning revealed that the patient had nursed a female patient from Turkey with laboratory confirmed measles one week before the start of her complaints.

Despite appropriate airborne isolation measures from the first moment this index patient had entered the hospital, she must have been the source of infection.

**Figure 2:**
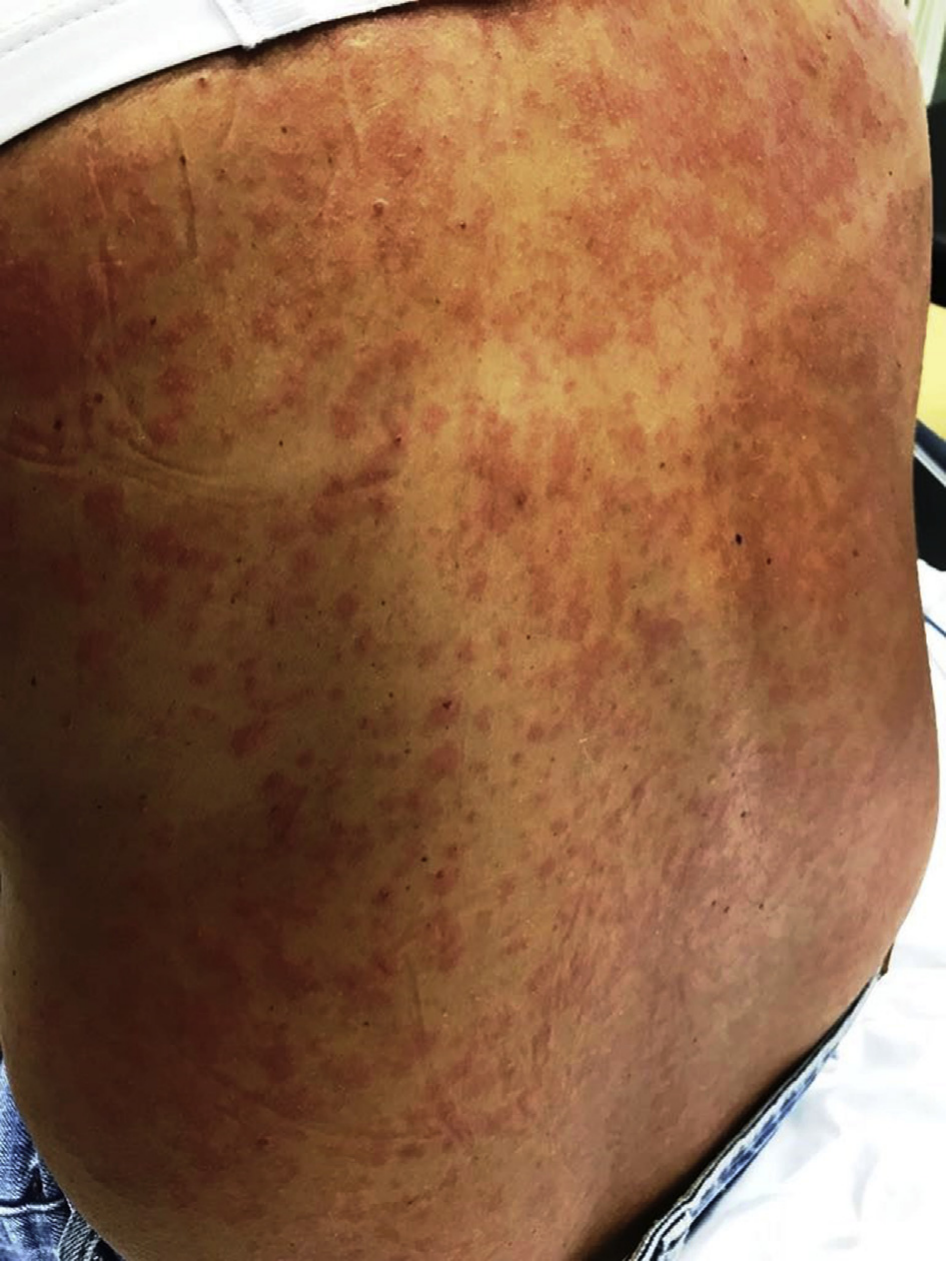
Maculopapular rash on the back of patient 2.

The second case was a 41-year-old female surgeon from Italy who presented herself at the emergency department of hospital B with fever, a new maculopapular rash (Figure 2) and Koplik spots on the buccal mucosa. She had just arrived in the Netherlands for a holiday. She had not been vaccinated for measles because this was not included in the national vaccination program during her childhood, and she was not aware of any contact with the virus. Our suspicion of measles was confirmed by anti-measles IgM antibodies and a positive PCR in plasma and nasopharyngeal swab. The patient was sent home and fully recovered after a total disease period of 21 days.

## Methods

In response to the first case, a questionnaire was developed in hospital B to assess protection against measles in HCP at risk to first encounter a patient with measles (doctors and nurses from the departments of emergency medicine, acute ward of internal medicine and ICU). Protection against measles was defined by a) born before 1965 [3], or b) twice vaccinated [4,5], or c) antibody titer >16.50 AU/ml.

## Results

The questionnaire was sent to 377 HCP of which 269 (71%) returned the questionnaire. Of these 269 responders, 42 (16%) were protected since they were born before 1965. One hundred and forty-eight (55%) HCP were protected since they had received two vaccinations (117 persons) or they were seropositive (20 persons) or had been through a measles infection before (11 persons). In total, 71% were protected against measles infection.

## Discussion

These two cases of HCP with measles infections emphasize the risk of nosocomial transmission of measles infection. The patient of the first case was even infected while proper isolation had been performed on the index patient.

Our survey demonstrates that still a considerable number of HCP are not protected against measles infection. This is in accordance with previously published data that show that a significant number (6.5–36%) of HCP in different areas of the world lack immunity against measles [6,7]. This may put HCP at risk during local outbreaks or when patients or professionals fly in from regions were measles transmission is present, as illustrated by our second case.

In our setting, we used different criteria for measles protection. Criterium A is based on the evidence that before the introduction of vaccination almost every child in The Netherlands was infected with measles before their fifth year [8]. People born before 1965 are therefore assumed to be immune. Criteria B was used because after two vaccinations, more than 99% of children develop immunity [4]. Criteria C, an antibody titer >16.50 AU/ml, is the most accurate criterium but is not known in every participant.

Our study has two limitations. First, only 71% of the personnel responded to the questionnaire. However, characteristics of responders and non-responders were comparable. Secondly, 20% of the responders did not know their own vaccination status and of those, only 25% participated in analysing their immune status. The main reason for not participating was that people had forgotten or were too busy. Therefore, of 40 responders the immune status is unknown and could be positive. This means that the protection coverage of 71% is possibly underestimated.

Suboptimal measles immunity in health care settings is an ongoing problem and interventions are necessary to prevent infection and transmission. Key components are awareness and registration of immunity of personnel at the time of employment and offering vaccination. In many places around the world, measles immunity is not routinely monitored and vaccination against measles is not offered to HCP [9]. These precautions would help to prevent healthcare-associated spread like the cases mentioned above and to minimize hospital outbreak-response costs [10].

## Conclusion

As illustrated by the two cases, health care professionals are at risk of measles infection. Current protection rate in our hospital is estimated at 71%. It is paramount for employee and patient health protection and for disease control to have an accurate registration of employee immunity and interventions to improve this immunity.

This article including the images was published with permission of both patients.

## Authors’ contributions

A.C. Westgeest: Writing – original draft

D. de Mooij: Writing – review & editing

C.Y. Eger: Writing – review & editing; Investigation

N.M. Delfos: Writing – review & editing

M. van der Feltz: Writing – review & editing

L.G. Visser Writing: – review & editing

G.H. Groeneveld: Writing – review & editing; Supervision
