# Erratum to “Measles outbreaks – potential threat for health care professionals” Infect Prev Pract, Volume 2, Issue 3, September 2020, 100074

**DOI:** 10.1016/j.infpip.2022.100208

**Published:** 2022-02-17

**Authors:** A.C. Westgeest, D. de Mooij, C.Y. Eger, N.M. Delfos, M. van der Feltz, L.G. Visser, G.H. Groeneveld

**Affiliations:** a Leiden University Medical Centre, Department of Infectious Diseases, C5-P, P.O. box 9600, 2300 RC Leiden, The Netherlands; b Alrijne Hospital Leiderdorp, Simon Smitweg 1, 2353 GA Leiderdorp, The Netherlands

The publisher regrets that the Informed Consent statement for this paper was not published in the final version. The relevant Informed Consent statement can be found below:

“**Informed consent**

This article including the images was published with permission of both patients.”

The publisher would like to apologise for any inconvenience caused.

